# Recurrent lower urinary tract infections: more than an infection for older women

**DOI:** 10.55730/1300-0144.5706

**Published:** 2023-05-09

**Authors:** Sencer GANİDAĞLI, Ercüment ÖZTÜRK, Zeynel Abidin ÖZTÜRK

**Affiliations:** Department of Internal Medicine, Division of Geriatric Medicine, Faculty of Medicine, Gaziantep University, Gaziantep, Türkiye Turkiye

**Keywords:** Recurrent lower urinary tract infections, comprehensive geriatric assessment, handgrip strength, older women

## Abstract

**Background/aim:**

Older adults tend to have more urinary tract infections (UTIs). The frequency of recurrent lower urinary tract infections (rLUTIs) increases with age. rLUTIs are associated with long-term chronic effects on geriatric syndromes in older adults. We aimed to investigate possible risk factors that influence rLUTIs in older adults based on comprehensive geriatric assessment (CGA).

**Materials and methods:**

This cross-sectional study included 235 older adults admitted to Gaziantep University’s Geriatric Outpatient Clinic between June 1 and November 30, 2022. All patients underwent CGA. The Geriatric Depression Scale (GDS), the European Quality of Life-Five Dimension (EQ-5D) questionnaire, the Pittsburgh Sleep Quality Index (PSQI), the Katz Index of Activities of Daily Living (ADL), the Lawton and Brody Index of Instrumental Activities of Daily Living (IADL), and the Mini Nutritional Assessment (MNA) tool were,administered. Handgrip strength (HGS) and gait speed were also measured, and the number of falls in the last year was recorded.

**Results:**

The mean age of the participants was 72.8 ± 6.8 years and 61.3% were female. Sixty-four patients had rLUTIs. The rLUTI group had higher frequencies of sarcopenia, hypertension, and diabetes; higher numbers of comorbidities and medications; higher GDS and PSQI scores; and more reported falls. They had lower ADL, MNA, EQ-5D, and gait speed scores. HGS was found to be lower in women with rLUTIs. Higher numbers of comorbidities and GDS scores and lower HGS were independent predictors of rLUTIs in women (p = 0.011, OR: 1.75; p = 0.018, OR: 1.14; and p = 0.042, OR: 0.91, respectively).

**Conclusion:**

We revealed that decreased HGS, higher GDS, and the number of comorbidities in older women were independent risk factors for rLUTIs. Our findings offer a new perspective on the importance of CGA in diagnosing and preventing rLUTIs.

## 1. Introduction

Older adults are susceptible to infections due to comorbidities and impaired immune function [1]. Lower urinary tract infection (LUTI), an infection of the bladder, is the most common infection among nursing home residents and the second most common infection among community-dwelling and hospitalized older adults [2,3]. Having a LUTI more than three times in a year or more than twice in 6 months is considered recurrent (rLUTI) [4,5]. Women tend to have more LUTIs than men due to anatomical differences, especially in terms of urethral length. It has been reported that 40%–50% of women have experienced a UTI in their lifetime, 10% of women over 65 have had one in the past 12 months, and 20%–30% of older women have rLUTIs [6,7]. The diagnosis of symptomatic LUTI requires the presence of urinary tract-specific symptoms, such as dysuria, urgency, suprapubic tenderness, and bacteriuria (≥10^5^ colony-forming bacterial units per milliliter) in a urine sample [8]. Older age, female sex, previous LUTI, urinary retention, and urinary incontinence are well-defined risk factors for LUTIs [8,9]. Urinary catheterization is recognized as another serious risk factor for older hospitalized patients [10].

Geriatric syndromes are common health conditions among older adults and increase the risk of morbidity and disability. Various studies have provided evidence about the relationship between LUTIs and geriatric syndromes, and treating geriatric syndromes, such as sarcopenia and malnutrition, is known to prevent LUTIs [11]. Sarcopenia increases the risk of infections in hospitalized patients, as well as in the community [12,13]. Hand grip strength (HGS), an important component of the diagnosis of sarcopenia, was found to decline in patients with lower urinary tract symptoms [14]. Likewise, polypharmacy, a common problem in older age due to increasing comorbidities, is a potential risk factor that negatively affects treatment efficiency in cases of LUTIs [6]. The prevalence of depression, the most common psychological disorder in older adults, has been reported at between 7% and 63% [15,16]. It impairs functionality, reduces quality of life, and increases mortality in older adults [17,18]. A recent study stated that LUTIs predispose older men to depressive symptoms [19]. Older adults are at risk for malnutrition due to age-related factors (physiological changes, increased number of comorbidities, polypharmacy, etc.). A study involving 188 older people living in residential care facilities showed that LUTIs were independently associated with poor nutritional status [20]. We hypothesized that rLUTIs have a negative effect on geriatric syndromes and our aim was to investigate the association between rLUTIs and geriatric syndromes based on comprehensive geriatric assessment (CGA).

## 2. Materials and methods

### 2.1. Study design and participants

This cross-sectional study included 235 individuals over the age of 65 who were admitted to Gaziantep University’s Geriatric Outpatient Clinic between June and November 2022. All participants underwent CGA. Patients with malignancy, renal impairment with glomerular filtration rate of <30 mL/min, chronic liver disease, acute or chronic infection, chronic inflammatory diseases, cognitive impairment, comorbidities that impair gait and balance (e.g., mobility, vision, and hearing disorders), and conditions that may affect bioelectrical impedance analysis measurements (e.g., amputation, edema, and severe fluid and electrolyte imbalance) were excluded from the study.

### 2.2. Comprehensive geriatric assessment

All participants underwent CGA, which included the assessment of sarcopenia, activities of daily living, instrumental activities of daily living, falls, cognitive and nutritional status, and depressive symptoms.

### 2.3. Diagnosis of rLUTI

The medical records of all patients were retrospectively analyzed for the past 1 year. Those who were diagnosed and treated for LUTIs three or more times in the last year or two or more times in the last 6 months were designated as experiencing rLUTIs [4,5].

### 2.4. Sarcopenia assessment

Sarcopenia was diagnosed according to the European Working Group on Sarcopenia in Older People (EWGSOP-2) diagnostic criteria for the presence of low muscle strength and low muscle mass. Muscle strength was measured with a Jamar hydraulic hand dynamometer (Tekzen, China) [21]. Threshold values for HGS were determined according to the values for older Turkish adults (22 kg for women and 32 kg for men) [22]. Muscle mass was measured using a Tanita SA165-A-0950U3 bioelectrical impedance analyzer (Tanita Corporation, Japan). Muscle mass was assessed by skeletal muscle mass index (SMMI) adjusted for body mass index (BMI). Values of less than 0.823 in women and 1.049 in men were considered low muscle mass [23]. In addition, muscle mass was calculated with the formula of fat-free mass (FFM) × 0.566, and values of <9.2 for men and <7.4 for women were considered to reflect low muscle mass. Sarcopenic patients with only low muscle strength were defined as having probable sarcopenia, those with low muscle strength and mass were defined as having confirmed sarcopenia, and those with confirmed sarcopenia and low physical performance were defined as having severe sarcopenia.

Gait speed is a significant parameter for assessing sarcopenia severity [21]. Values below 0.8 m/s reflect low physical performance, which is associated with poor outcomes, increased mortality, and disability [24]. Gait speed was recorded as the time required to walk 4 m.

### 2.5. Sleep quality assessment

The Pittsburgh Sleep Quality Index (PSQI) was used to evaluate sleep quality. It is a validated self-rated questionnaire that assesses sleep quality and sleep disturbance over a 1-month period [25]. The PSQI evaluates seven sleep domains: quality, latency, duration, efficiency, disturbances, use of sleep medication, and daytime dysfunction. The sum of the domains’ scores ranges from 0 to 21, and a score of >5 is considered to reflect poor sleep quality [26].

### 2.6. Geriatric Depression Scale (GDS)

The GDS is a questionnaire consisting of 30 questions used to evaluate depressive symptoms in older individuals. Positive answers are coded as 0 and negative answers as 1. A score of 0–10 is classified as signifying no depression, a score of 11–13 as possible depression, and a score of ≥14 as depression [27,28].

### 2.7. Assessment of cognitive function

The Turkish version of the Mini Mental State Examination (MMSE), a 30-point questionnaire, was used to evaluate the cognitive functions of the participants [29]. The MMSE examines five areas of cognition: language (9 points), orientation (10 points), registration (3 points), memory (3 points), and attention and calculation (5 points). The Turkish version was validated and the cut-off point was found to be 23/24 for the diagnosis of mild dementia in the Turkish population [29].

### 2.8. Activities of daily living

The Katz Index of Activities of Daily Living (ADL) and the Lawton and Brody Index of Instrumental Activities of Daily Living (IADL) were used to assess the participants’ dependency in performing activities of daily living.

The ADL assesses a person’s ability to perform routine daily activities independently, such as bathing, dressing, using the toilet, transferring, continence, and feeding. Total scores range from 0 to 6 and higher scores indicate greater independence [30].

The IADL measures independence in more specific daily tasks, such as shopping, housekeeping, doing laundry, food preparation, using the telephone, using transportation, taking medicine, and managing money. The total score ranges from 0 to 8 and higher scores indicate greater independence [31].

### 2.9 Quality of life assessment

Quality of life was assessed with the European Quality of Life-5 Dimensions (EQ-5D) scale. Its five dimensions are mobility, self-care, usual activities, pain/discomfort, and anxiety/depression, and the EQ-5D index score is calculated according to the answers given. An index score of 1 indicates flawless health and negative values indicate that someone is dependent, bedridden, or unconscious [32].

### 2.10. Number of falls

Participants were asked to report the number of falls they had experienced in the previous 12 months.

### 2.11. Mini Nutritional Assessment (MNA) tool

The MNA is a screening and assessment tool for nutritional status. A score of ≥24 indicates adequate nutritional status, 17–23.5 indicates malnutrition risk, and <17 indicates malnutrition [33].

### 2.12. Polypharmacy

Polypharmacy was defined as the concomitant use of five or more medications [34].

### 2.13. Statistical analysis

Statistical analyses were performed with IBM SPSS 22.0 for Windows (IBM Corp., USA). The normality of the distribution was checked using the Shapiro–Wilk test. We used the independent samples t-test and Mann–Whitney U test to compare two sets of independent variables and the chi-square test to evaluate relationships between categorical variables. Multivariate logistic regression analysis was used to identify independent predictors of rLUTIs separately for male and female sex. A p-value of less than 0.05 was considered statistically significant. A variance inflation factor (VIF) of >5 was regarded as indicating a collinearity problem between the variables.

## 3. Results

A total of 235 individuals with a mean age of 72.8 ± 6.8 years were included in this study and 61.3% were women. Of the participants, 64 (27.2%) had a history of rLUTIs. There was no statistically significant difference between the groups with and without rLUTIs in terms of age, presence of diabetes and polypharmacy, or SMMI. The rLUTI group had a higher frequency of sarcopenia; higher numbers of comorbidities, medications, and falls; and higher GDS and PSQI scores. They had lower ADL, MNA, and EQ-5D index scores and gait speed. HGS was also lower among women with rLUTIs. Table 1 shows the sociodemographic characteristics and CGA analysis results of the participants. In patients with rLUTIs, the frequency of probable and severe sarcopenia was higher (p = 0.008 and p = 0.005, respectively), while there was no statistically significant difference in the frequency of confirmed sarcopenia between the groups (as shown in Table A1 in the Appendix).

The VIF was calculated and the variables of SMMI, FFM, and EQ-5D (VIF = 2.1, 2.3, and 2.6, respectively) were excluded from the models due to collinearity. Multivariate logistic regression analysis was performed for men and women separately and showed that a higher number of comorbid diseases, higher GDS scores, and lower HGS were independent predictors of rLUTIs in women (p = 0.011, OR: 1.75; p = 0.018, OR: 1.14; and p = 0.042, OR: 0.91, respectively) (Table 2). There were no independent risk factors for rLUTIs in men (Table 3).

## 4. Discussion

The present study showed that a higher number of diseases and GDS scores and lower HGS were predictive risk factors for rLUTIs in older women. Numerous studies in the medical literature have investigated the relationship between lower urinary tract symptoms (nocturia, overactive bladder, and urinary incontinence) and common geriatric conditions, but few studies have investigated relationships between rLUTIs and geriatric syndromes. Recurrent UTIs are more significant than nonrecurrent cases because they have long-term negative effects on health. For instance, depression and mental stress were found more frequently in cases of recurrent UTIs [35]. In addition, rLUTIs negatively affect the economic domain of quality of life in postmenopausal women and the economic, physical, and psychosocial domains in men [36,37].

In our study, approximately one of every three women had rLUTIs. Consistent with our results, previous studies have shown that 20%–30% of women have rLUTIs [7,38]. The prevalence of rLUTIs increases with age due to various age-related conditions, such as sarcopenia, which affects all skeletal muscle groups, including the pelvic floor muscles. One previous study reported the relationship between sarcopenia and pelvic floor dysfunction in women and emphasized the importance of evaluating patients with pelvic floor dysfunction for sarcopenia [39]. Pelvic floor muscles are important for maintaining continence, and weakness of the pelvic floor muscles may result in incontinence [40]. Moreover, Hu et al. showed that urinary incontinence may increase the risk of urinary infections [41]. Another study by Erdoğan et al. showed that urinary incontinence was independently associated with sarcopenia [42]. The findings of these studies may be interpreted as pointing to an indirect relationship between sarcopenia and UTIs. For example, in one study, UTI and in-hospital morbidity were found to be related to sarcopenia [43]. Although those results were similar to ours, that study was carried out with hospitalized patients and all UTIs were catheter-associated. A recent study conducted by Zhang et al. that included a total of 2512 patients showed that UTIs increased sarcopenia 1.7-fold in diabetic patients [13]. That study was carried out only with diabetic patients and it assessed the effect of UTIs on sarcopenia. A study that included female participants with a mean age of 48.6 revealed that low HGS was an independent variable predicting urinary tract symptoms such as nocturia, incontinence, and overactive bladder [14]. According to a cohort study with a mean age of 80.1 years, although low gait speed was an independent risk factor for severe overactive bladder, HGS was not [44]. However, gait speed was significantly lower in the rLUTI group in our study, and multivariate logistic regression analysis showed that it was not a risk factor for rLUTIs. In addition, according to our findings, the frequency of patients with probable and severe sarcopenia was higher in patients with rLUTIs, although the frequency of patients with confirmed sarcopenia was not higher. This finding may be a consequence of the small number of patients with confirmed sarcopenia in our study.

In the current study, depression was found to be an independent risk factor for rLUTIs in women. Many studies have noted the negative impact of UTIs on depression. This is to be expected, because UTIs can cause chronic inflammation and pain that contribute to the development of depression [45,46]. Melotti et al. inferred that overactive bladder and nocturia, common symptoms of LUTIs, were associated with the severity of depression [47]. Similarly, our findings indicated that the mean GDS score was higher in the rLUTI group. Moreover, a prospective, multicenter observational study reported that patients diagnosed with UTIs who received oral prophylactic treatment for 3 months had a 25% decrease in depression scores at the end of 6 months [48]. LUTIs have been shown to be risk factors for depression in previous studies. Our study has shown that depression is an independent predictor of rLUTIs in women. One recent study demonstrated that depression diminished the chemotactic function of both neutrophils and lymphocytes [49], while another determined that patients with severe depression were 3.7 times likelier to be medication nonadherent than nondepressed patients [50]. We considered that depression increased the risk of UTIs by increasing the tendency to experience UTIs, causing medication nonadherence in UTI treatment, and increasing the risk of UTIs due to the side effects of antidepressants used in treatment. We suggest that every depressive older woman be assessed in terms of rLUTI.

As is known, malnutrition is associated with impaired immunity, which increases the risk of infections [51]. In addition, a previous study reported that LUTI is a risk factor for malnutrition [52]. In concordance with the literature, the present study indicated that nutrition status was poorer in the rLUTI group. In addition, patients with rLUTIs had poorer sleep quality in our study. As previous studies have reported, this is thought to be the result of lower urinary tract symptoms such as nocturia, pollakiuria, and dysuria, which impair sleep quality [53].

The prevalence of polypharmacy varies between 26.3% and 39.9% in the older population [54]. In our study, 54.5% of the participants had polypharmacy. Another significant finding of our study was that the number of medications and comorbidities was higher among patients with rLUTIs. It was also noted in a cross-sectional study that the number of drugs used was identified as a risk factor for bladder dysfunction [6]. The impact of comorbidities,such as diabetes mellitus, hypertension, and cerebrovascular disease and/or medications such as diuretics, antipsychotics, antidepressants, anticholinergics, calcium channel blockers, and alpha-adrenergic agonists on bladder function may lead to the development of rLUTIs [55]. Likewise, multimorbidity is a factor that increases susceptibility to infection. In our study population, hypertension, coronary artery disease, asthma/COPD, and depression were more common in the rLUTI group. Therefore, patients with a higher number of comorbidities may have been exposed to higher numbers of medications that affect bladder function and may have had an increased risk of rLUTIs.

We found no statistical differences between the two groups in terms of the frequency of benign prostate hypertrophy or diabetes. This finding may be a result of the small number of participants. In addition, there was a significant difference between the two groups in terms of the rates of coronary artery disease and hypertension.

The limitations of this study were its cross-sectional nature and the small number of male participants with rLUTIs. On the other hand, our study’s strengths include, first of all, the fact that our findings were derived from the CGA of older adults, and this permitted an evaluation of the relationship between rLUTIs and common geriatric syndromes. Second, unlike other studies assessing the relationships between LUTI and geriatric syndrome diseases separately, our study is unique in evaluating rLUTI and its relationships with common geriatric syndromes.

In conclusion, our study showed that lower HGS, higher GDS scores, and higher numbers of comorbidities were independent predictors of rLUTIs in older women. Based on our findings, we suggest that CGA should be an indispensable part of geriatric examinations in all older adults. In light of our results, we conclude that measuring HGS and determining depressive symptoms can help identify patients at risk for rLUTI. This study offers a new perspective on identifying the risk factors for rLUTI that may be helpful in the prevention of rLUTI and its associated complications. Further studies are needed to investigate how sarcopenia, depression, and other diseases cause rLUTIs.

## Figures and Tables

**Table 1:** Participants’ sociodemographic characteristics and comprehensive geriatric assessment results (n = 235).

Variables		rLUTI (−) (n=171)	rLUTI (+) (n=64)	p	Total (n=235)
Sex					
Female		93 (54.4%)	51 (79.7%)	<0.001*	144 (61.3%)
Male		78 (45.6%)	13 (20.3%)		91 (38.7%)
Age †		72.8 ± 6.7	72.9 ± 6.9	0.879	72.8 ± 6.8
Number of comorbidities #		3 (2)	4 (2)	<0.001*	3 (2)
Number of medications #		5 (4)	5.5 (4)	0.048*	5 (4)
Comorbidities					
Hypertension		94 (55.0%)	51 (79.7%)	0.001*	145 (61.7%)
Diabetes mellitus		91 (53.2%)	41 (64.1%)	0.136	132 (56.2%)
Coronary artery disease		43 (25.1%)	26 (40.6%)	0.020*	69 (29.4%)
Asthma/COPD		26 (15.2%)	17 (26.6%)	0.045*	43 (18.3%)
BPH		16 (9.4%)	6 (9.4%)	0.997	22 (9.4%)
Depression		51 (29.8%)	29 (45.3%)	0.003*	80 (34%)
Polypharmacy		86 (50.9%)	41 (64.1%)	0.071	127 (54.5%)
ADL #		6 (1, 1–6)	5(2, 1–6)	<0.026*	6 (1, 1–6)
IADL#		7 (4, 0–8)	6 (5, 0–8)	0.121	7 (4, 0–8)
GDS #		8 (10, 0–30)	13(8, 2–30)	<0.001*	10 (0–30)
MNA #		23.5 (10, 10–30)	21.5 (7.5, 8–29)	0.021*	23.0 (9.5, 8–30)
EQ-5D #		0.67 (0.58, −0.21–1.00)	0.35 (0.45, −0.53–1)	<0.001*	0.53 (0.59, −0.53–1.00)
PSQI #		3 (2, 0–5)	8 (4, 5–17)	<0.001*	6 (5, 0–17)
Sarcopenia		52 (30.4%)	32 (50.0%)	0.006*	84 (35.7%)
HGS (kg) †	Female	18.6 ± 7.4	16.2 ± 4.9	0.044*	17.7 ± 6.7
	Male	26.6 ± 6.7	28.0 ± 7.4	0.528	26.8 ± 7.7
SMMI (BMI) †	Female	0.81 ± 0.13	0.77 ± 0.08	0.067	0.79 ± 0.12
	Male	1.15 ± 0.15	1.21 ± 0.19	0.232	1.16 ± 0.15
Gait speed (m/s) †		0.82 ± 0.29	0.70 ± 0.28	0.007*	0.79 ± 0.29
Number of falls #		0 (1, 0–10)	2.5 (6, 0–10)	0.020*	0 (1.75, 0–10)

* p < 0.05;

† Data are presented as mean ± SD.

# Data are presented as median (interquartile range, min-max).

rLUTI: Recurrent lower urinary tract infection; COPD: chronic obstructive pulmonary disease; BPH: benign prostatic hyperplasia; ADL: Katz Index of Activities of Daily Living; IADL: Lawton & Brody Index of Instrumental Activities of Daily Living; GDS: Geriatric Depression Scale; MNA: Mini Nutritional Assessment tool; EQ-5D: European Quality of Life-5 Dimensions Questionnaire; PSQI: Pittsburgh Sleep Quality Index; HGS: handgrip strength; SMMI: skeletal muscle mass index.

**Table 2:** Multivariate logistic regression analysis results of the independent variables for recurrent lower urinary tract infection (rLUTI) in female participants.

	rLUTI	
Variable	OR [95% CI]	p
• Age	1.05 [0.96–1.14]	0.274
• Number of diseases	1.75 [1.14–2.70]	0.011*
• Number of medications	0.88 [0.66–1.16]	0.360
• ADL	0.79 [0.45–1.38]	0.412
• GDS	1.14 [1.02–1.26]	0.018*
• MNA	1.02 [0.93–1.13]	0.651
• PSQI	1.02 [0.82–1.20]	0.862
• HGS	0.91 [0.80–0.99]	0.042*
• Gait speed	0.30 [0.33–2.74]	0.286

* p < 0.05 according to multivariate binary logistic regression analysis.

CI: Confidence interval; OR: odds ratio; ADL: Katz Index of Activities of Daily Living; GDS: Geriatric Depression Scale; MNA: Mini Nutritional Assessment tool; PSQI: Pittsburgh Sleep Quality Index; HGS: handgrip strength.

**Table 3:** Multivariate logistic regression analysis results of the independent variables for recurrent lower urinary tract infection (rLUTI) in male participants.

	rLUTI	
Variable	OR [95% CI]	p
Age	1.00 [0.87–1.15]	0.971
Number of diseases	1.60 [0.81–3.16]	0.176
Number of medications	0.69 [0.89–1.07]	0.116
ADL	0.66 [0.31–1.39]	0.274
GDS	0.90 [0.73–1.12]	0.353
MNA	0.89 [0.77–1.04]	0.153
PSQI	1.18 [0.93–1.49]	0.181
HGS	1.10 [0.97–1.25]	0.145
Gait speed	1.71 [0.07–40.3]	0.738

* p < 0.05 according to multivariate binary logistic regression analysis.

CI: Confidence interval; OR: odds ratio; ADL: Katz Index of Activities of Daily Living; GDS: Geriatric Depression Scale; MNA: Mini Nutritional Assessment tool; PSQI: Pittsburg Sleep Quality Index; HGS: handgrip strength.

**Table A1:** Associations between probable, confirmed, and severe sarcopenia and recurrent lower urinary tract infection (rLUTI).

	rLUTI (−)	rLUTI (+)	p	Total
Probable sarcopenia	60 (53.1%)	13 (29.5%)	0.008*	73 (46.5%)
Confirmed sarcopenia	23 (20.4%)	9 (20.5%)	0.989	32 (20.4%)
Severe sarcopenia	30 (26.5%)	22 (50%)	0.005*	52 (33.1%)

* p < 0.05
